# Single-strain and consortium inoculations with plant-beneficial *Pseudomonas* spp. promote lettuce growth under field conditions

**DOI:** 10.1128/spectrum.03509-25

**Published:** 2025-12-30

**Authors:** Adrien Biessy, Mélanie Cadieux, Florence Mc Duff, Arianne Deshaies, Kosal Khun, Joël Lafond-Lapalme, Philippe Vigneault, Martin Filion

**Affiliations:** 1 Department of Plant Science, McGill University, Macdonald Campus 151165, Sainte-Anne-de-Bellevue, Quebec, Canada; 2 Saint-Jean-sur-Richelieu Research and Development Centre, Agriculture and Agri-Food Canada, Saint-Jean-sur-Richelieu, Quebec, Canada; Department of Environmental Sciences, Connecticut Agricultural Experiment Station, New Haven, Connecticut, USA

**Keywords:** plant growth-promoting rhizobacteria, *Pseudomonas protegens*, *Pseudomonas putida*, qPCR, *Pseudomonas *consortium, unmanned aerial vehicle (UAV)

## Abstract

Plant-beneficial *Pseudomonas* spp. can promote plant growth by a wealth of mechanisms. Developing microbial inoculants encompassing multiple *Pseudomonas* strains (consortia) could represent an attractive solution to some of the problems encountered by single-strain inoculants, such as inconsistent rhizosphere colonization under field conditions. In this study, we evaluated the potential of *Pseudomonas protegens* B21-024 and *Pseudomonas putida* B21-029, inoculated alone or in combination, to promote lettuce growth under greenhouse and representative commercial field conditions. Plant growth metrics, including shoot fresh and dry weights, were used to evaluate plant growth promotion. In addition, unmanned aerial vehicle imaging was used to monitor plant growth in the field. Strain-specific primer-probe sets were also developed to study, using the quantitative polymerase chain reaction, the population dynamics of the two strains in rhizosphere soil. *P. putida* B21-029 and the *Pseudomonas* consortium significantly increased lettuce shoot fresh/dry weight under greenhouse conditions. In the field, only inoculation with *P. protegens* B21-024 and the *Pseudomonas* consortium promoted lettuce growth by significantly increasing shoot dry weight. Interestingly, the nitrogen-rich bacterial growth medium used to prepare the inocula played a role in the plant growth promotion achieved. Overall, the rhizosphere population of both *Pseudomonas* strains remained relatively stable during the growing season. However, the population of *P. protegens* B21-024 was significantly higher when it was co-inoculated with *P. putida* B21-029 than when inoculated alone, suggesting the existence of a beneficial relationship. These findings support the use of *Pseudomonas* consortia to promote the growth of leafy greens under field conditions.

**IMPORTANCE:**

Some bacteria belonging to the genus *Pseudomonas* can establish mutually beneficial relationships with many crop species, resulting in disease suppression and/or plant growth promotion. These microorganisms show potential to replace, at least in part, synthetic fertilizers and pesticides in agricultural field settings. While some *Pseudomonas* strains have been shown to promote lettuce growth, successfully developing *Pseudomonas* consortia would have many advantages over single-strain inoculations, including better effectiveness and rhizosphere colonization. In this study, a consortium encompassing two *Pseudomonas* strains successfully promoted lettuce growth under greenhouse and field conditions. The nitrogen-rich bacterial growth medium used for the preparation of inocula contributed to the plant growth promotion achieved. Strain-specific molecular markers were also developed to monitor the abundance of each strain in rhizosphere soil throughout the growing season. The results obtained in this study indicate that the two *Pseudomonas* strains under study successfully colonized the lettuce rhizosphere.

## INTRODUCTION

Members of the genus *Pseudomonas* are rod-shaped Gram-negative Gammaproteobacteria that are found in many environments, including soil, water, and higher organisms (1). This large and diverse genus includes more than 300 species, some of which can cause diseases to plants and animals (2). For example, *P. syringae* is one of the most devastating bacterial plant pathogens to roam the phyllosphere (3, 4). By contrast, many *Pseudomonas* species and strains establish mutually beneficial relationships with a myriad of plant species, including many important crops (5–7). Plant-beneficial *Pseudomonas* spp. aggressively colonize the rhizosphere—the zone under the influence of the roots—and use root exudates as source of carbon and energy (5, 8, 9). In return, these microorganisms often protect the plant root system against soilborne plant pathogens (5, 7, 10), help the plant to cope with abiotic stresses (11), and/or directly promote plant growth (12).

Direct plant growth promotion by plant-beneficial *Pseudomonas* spp. is mediated by a wealth of mechanisms that result in biomass and/or yield increases. One of the main mechanisms used by plant-beneficial *Pseudomonas* spp. is the modulation of the plant hormonal balance (13). For example, many *Pseudomonas* strains produce phytohormones, such as auxins and gibberellins (14–16). Production of the auxin molecule indole-3-acetic acid (IAA) by plant-beneficial *Pseudomonas* spp. has been shown to increase root biomass and to promote the development of the root system, either by stimulating primary root elongation or by increasing root hair formation (13, 15). Conversely, gibberellic acid production increases shoot length and shoot fresh/dry weight (14). Moreover, many *Pseudomonas* strains produce the 1-aminocyclopropane-1-carboxylate (ACC) deaminase enzyme that degrades ACC, the precursor of the stress hormone ethylene (17–19). This enables the bacteria to act as a sink for ACC and to modulate endogenous ethylene levels, promoting root elongation and increasing biomass (17). Additional plant growth promotion mechanisms include phosphate solubilization (20, 21), and the production of volatile organic compounds with plant growth promotion activity (22, 23). By promoting the development of the root system, plant-beneficial *Pseudomonas* spp. improve the ability of the plant to absorb nutrients, thereby accelerating its growth and increasing crop yield.

Lettuce (*Lactuca sativa* L., Asteraceae) is an important horticultural crop that is grown in most areas worldwide. Growers are interested in new sustainable methods to increase yield and enhance lettuce nutritional quality. Many *Pseudomonas* strains have been shown to enhance lettuce growth under both controlled and field conditions (24–28). For example, Cipriano et al. reported that single-strain inoculations with *Pseudomonas* spp. promoted lettuce growth under field conditions (24). Over the years, the development of microbial inoculants encompassing multiple strains (microbial consortia) has been increasingly recognized as an attractive solution to some of the problems of single-strain inoculants, namely insufficient rhizosphere colonization and inconsistent plant growth promotion/disease suppression (10, 29–31). However, *Pseudomonas* spp. are known for their ability to produce a wide diversity of secondary metabolites with antimicrobial activity (32), which complexifies their successful association with other bacteria. For example, *Pseudomonas* strains producing the antibiotic 2,4-diacetylphloroglucinol (DAPG) inhibit the growth of bacteria from two major plant-beneficial genera, *Bacillus* and *Azospirillum* (28, 33, 34). Combining two or more *Pseudomonas* strains could potentially reduce the risk of incompatibility between bacterial strains during co-inoculation.

In a previous study, we showed that inoculation with the plant-beneficial strain *Pseudomonas protegens* B21-024 promoted lettuce growth under field conditions (27). This strain significantly increased lettuce shoot fresh/dry weight under optimal nitrogen conditions. In addition, inoculation with *P. protegens* B21-024 compensated for most of the yield losses caused by reduced nitrogen fertilizer application. Based on these promising results, co-inoculating *P. protegens* B21-024 with other *Pseudomonas* strains appears as an attractive strategy that could further improve lettuce growth.

In this study, we evaluated the ability of two *Pseudomonas* strains, *P. protegens* B21-024 and *Pseudomonas putida* B21-029, to promote lettuce growth and yield. First, we validated the plant growth promotion ability of the two strains, alone and in combination, under greenhouse conditions. Second, we carried out a field experiment to validate the plant growth promotion activity of the two strains, alone and in combination, under representative commercial field conditions. We used a combination of weekly manual plant sampling and biweekly unmanned aerial vehicle (UAV) flights to monitor lettuce growth. Finally, we designed two strain-specific primer-probe sets to quantify, using quantitative polymerase chain reaction (qPCR), the abundance of the two bacterial strains in the rhizosphere soil surrounding lettuce plants throughout the growing season.

## RESULTS

### Lettuce growth promotion under greenhouse conditions

**Fig 1:**
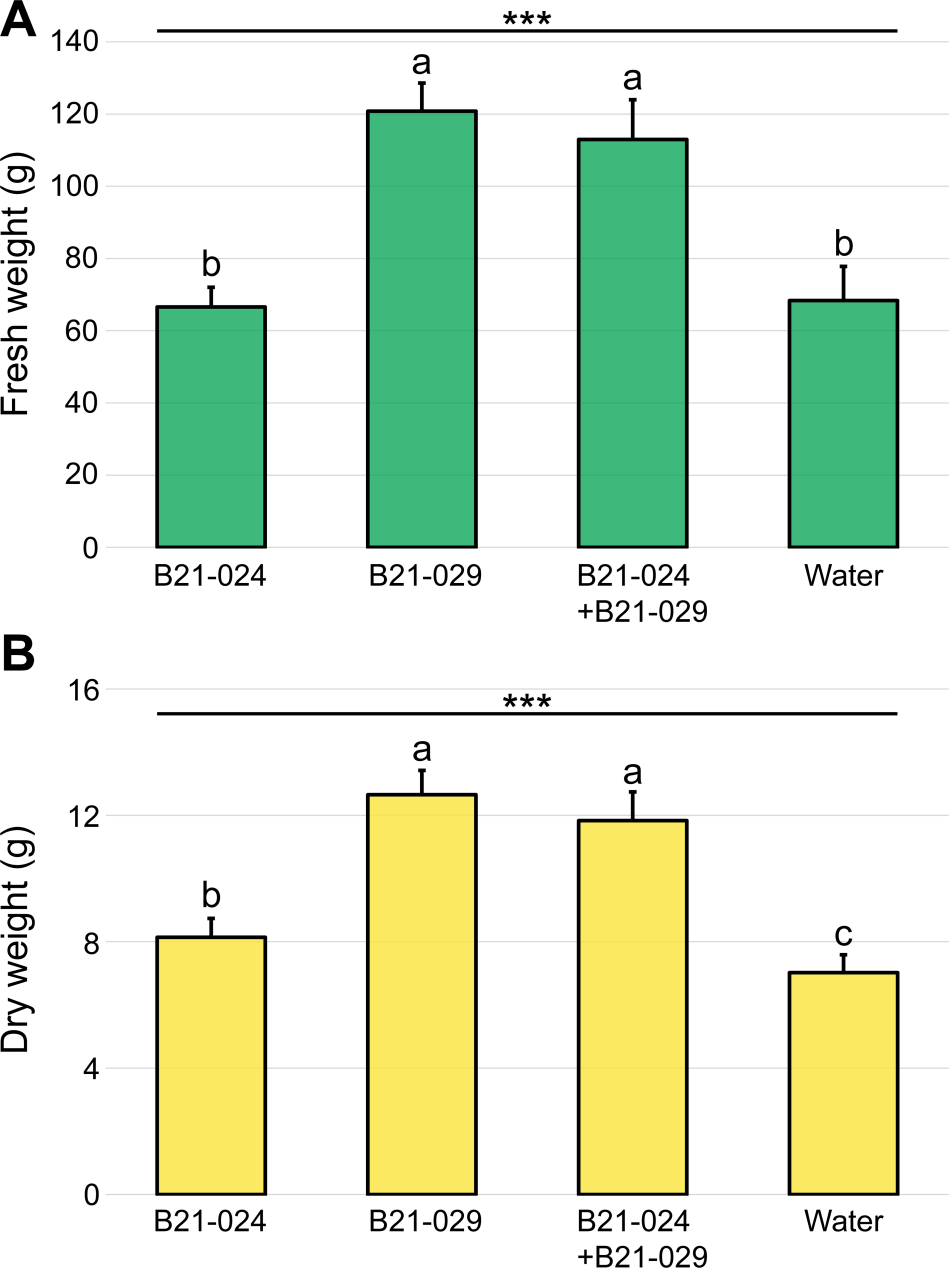
Plant growth promotion under greenhouse conditions. Lettuce seedlings were inoculated 13 days after sowing with four different treatments: three bacterial treatments (*P. protegens* B21-024, *P. putida* B21-029, and both *Pseudomonas* strains in combination) and a water control. Fifty-five days after sowing, the plants were harvested, and the shoot fresh weight (**A**) and shoot dry weight (**B**) were measured. The values are presented as means + standard errors. The three asterisks indicate a *P* value inferior to 0.001 (Friedman test). Treatments with different letters are significantly different according to Fisher’s LSD post hoc test (*α* = 0.05).

As a first step to evaluating the plant growth promotion potential of *P. protegens* B21-024 and *P. putida* B21-029, alone and in combination, a greenhouse experiment was carried out over a 55-day period. The inoculation treatments included i) *P. protegens* B21-024, ii) *P. putida* B21-029, iii) the two *Pseudomonas* strains in combination, and iv) a water control. *P. putida* B21-029 and the *Pseudomonas* consortium (B21−024+B21-29) significantly increased lettuce aboveground fresh biomass by 77% and 65%, respectively, when compared to the water-inoculated lettuce plants (Fig. 1). In addition, both treatments significantly increased shoot dry weight, with increases ranging from 69% to 80%. While inoculation with *P. protegens* B21-024 alone did not significantly increase aboveground fresh biomass (Fig. 1), this treatment significantly increased shoot dry weight by 16%.

### Lettuce growth promotion under field conditions

**Fig 2:**
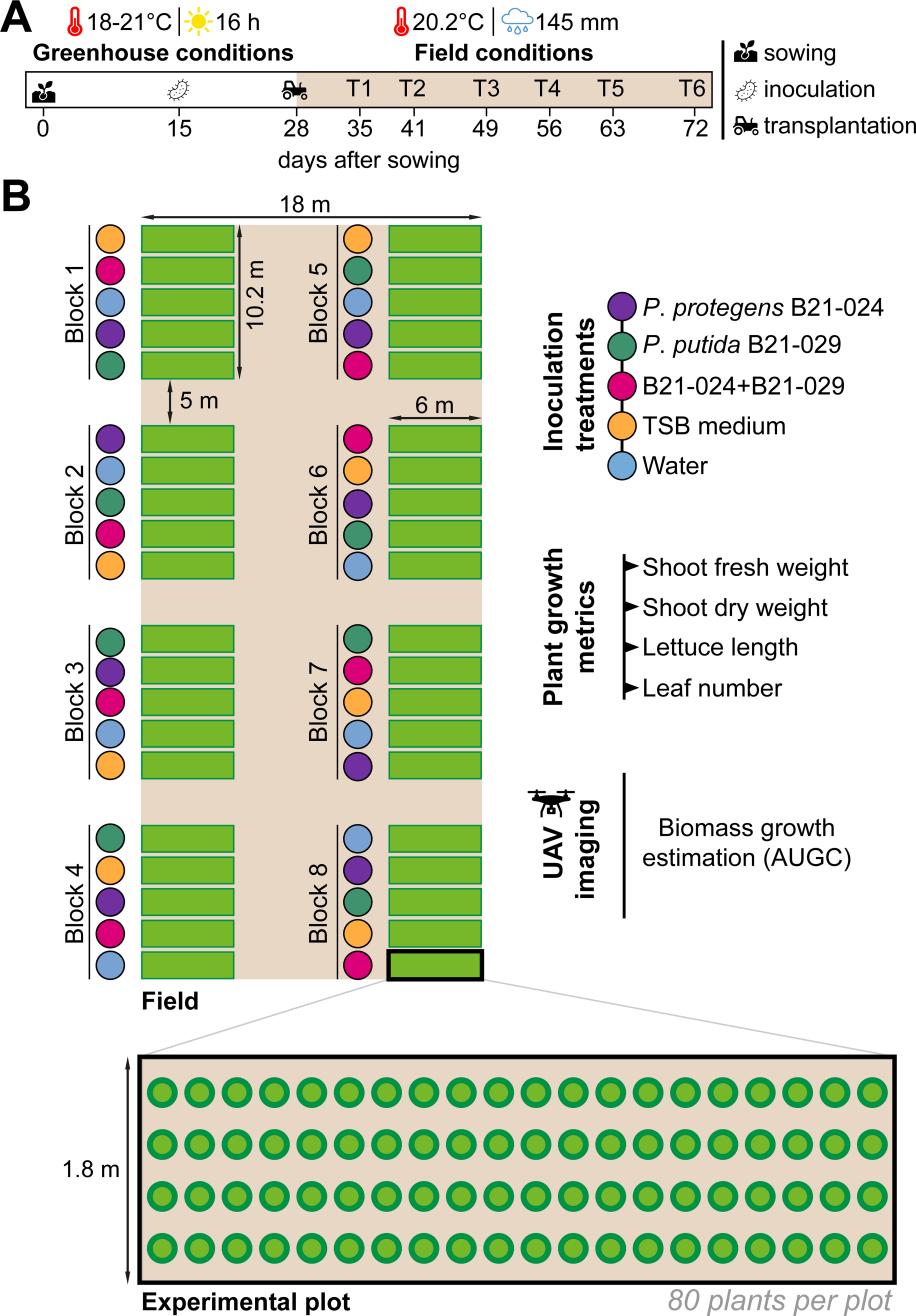
Timeline and experimental design of the field trial. (**A**) Romaine lettuce seeds were germinated and grown under greenhouse conditions until field transplantation. The seedlings were inoculated 15 days after sowing, and the plants were transplanted into the field 28 days after sowing. During the 44 days the lettuce plants were in the field, the average temperature was 20.2°C and total precipitation amounted to 145 mm. Lettuce plants were harvested at six different sampling times during the growing season: 35 (T1), 41 (T2), 49 (T3), 56 (T4), 63 (T5), and 72 (T6) days after sowing. (**B**) A randomized complete block design was used, with a total of eight blocks. The five inoculation treatments were randomized within each block, and each experimental plot was inoculated with the same treatment. The plots included a total of 80 lettuce plants each. One randomly selected lettuce plant per plot was sampled during the five intermediate samplings (T1, T2, T3, T4, and T5), whereas five lettuce plants per plot were harvested at the final sampling (T6). Four plant growth metrics were collected in this study: shoot fresh weight, shoot dry weight, lettuce length, leaf number. In addition, the growth of each individual lettuce was monitored throughout the growing season thanks to biweekly flights of an UAV mounted with imaging sensors. The area under the growth curve (AUGC) of the surface area metric was chosen to estimate aboveground biomass accumulation.

A field experiment was carried out in the summer of 2024 to test the plant growth promotion effect of *P. protegens* B21-024 and *P. putida* B21-029, alone and in combination, under representative commercial field conditions (Fig. 2). Five treatments were used: three bacterial treatments (*P. protegens* B21-024, *P. putida* B21-029, and a consortium of these two strains), a tryptic soy broth (TSB) treatment, and a water control treatment. The TSB treatment was added as the two *Pseudomonas* strains were initially grown in TSB medium, and fresh TSB medium was added during the preparation of the inocula (one-third of the total volume) to support the establishment of the strains in the rhizosphere. In addition, this treatment previously promoted the growth of field-grown lettuce plants under limited nitrogen availability conditions (27). Therefore, the use of this treatment enabled us to monitor whether the TSB medium promotes plant growth and determine to what extent. Lettuce plants were harvested at six different sampling times during the growing season: 35 (T1), 41 (T2), 49 (T3), 56 (T4), 63 (T5), and 72 (T6) days after sowing. One randomly selected lettuce plant per plot was sampled during the five intermediate samplings (T1:T5; 8 per treatment), whereas five lettuce plants per plot were harvested at the final sampling (T6; 40 per treatment). Four plant growth metrics were measured: shoot fresh weight, shoot dry weight, lettuce length, and leaf number.

**Fig 3:**
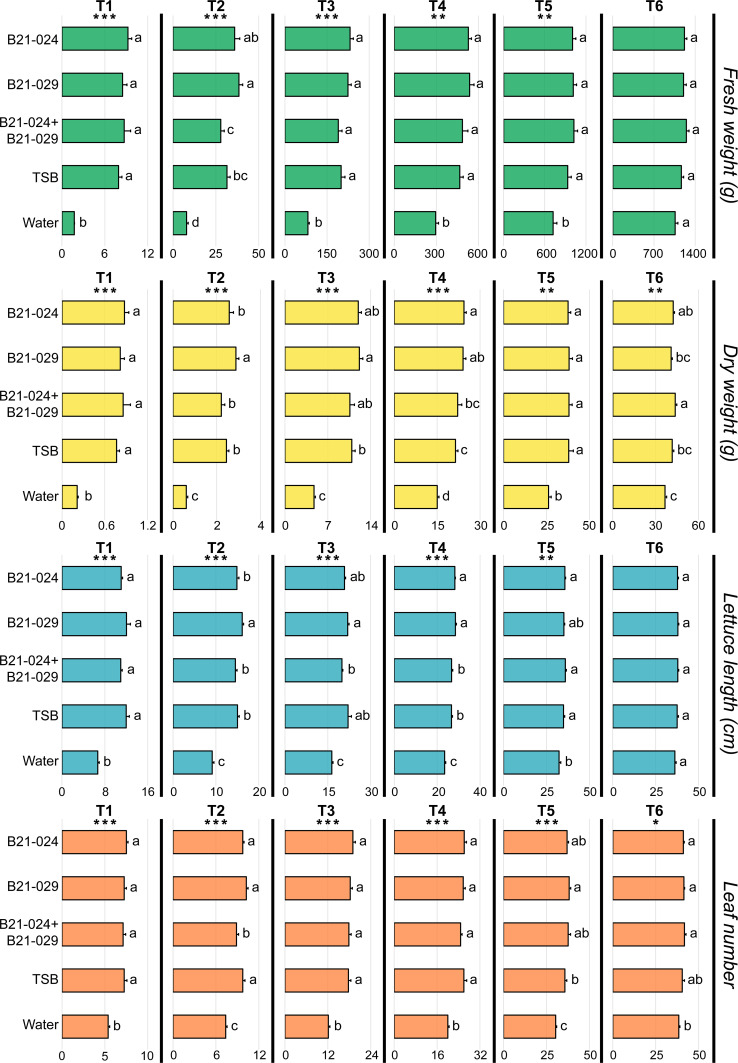
Plant growth promotion under field conditions. Lettuce plants were grown from seeds in a greenhouse and inoculated with five different treatments: *P. protegens* B21-024, *P. putida* B21-029, a combination of both *Pseudomonas* strains, TSB medium, and water. The plants were transplanted into the field 28 days after sowing. Lettuce plants were harvested at six different sampling times throughout the growing season: 35 (T1), 41 (T2), 49 (T3), 56 (T4), 63 (T5), and 72 (T6) days after sowing. Four plant growth metrics were measured: shoot fresh weight, shoot dry weight, lettuce length, and leaf number. The values are presented as means + standard errors. The five treatments were compared to each other at each sampling time independently. One, two, or three asterisks refer to *P* values inferior to 0.05, 0.01, and 0.001, respectively (Friedman test). Treatments with different letters are significantly different from each other according to Fisher’s LSD post hoc test (*α* = 0.05).

During most of the growing season (T1–T5), lettuce plants inoculated with one of the three bacterial treatments or with the TSB treatment had a higher aboveground biomass (fresh and dry weight) and a larger number of leaves than water-inoculated lettuce plants (Fig. 3). Most treatments also increased lettuce length when compared to the water control at the five intermediate samplings (T1–T5). The plant growth promotion effect of these four treatments was remarkably high at the first sampling times but gradually decreased as the growing season progressed (Fig. 3; Fig. S1). For example, at the first sampling time (35 days after sowing), the fresh biomass of lettuce plants inoculated with one of the three bacterial treatments or with the TSB treatment was, on average, more than four times greater than the fresh biomass of water-inoculated plants. Sixty-one days after sowing (T5), the differences between these four treatments and the water treatment ranged from 29% to 42%. While similar plant growth promotion was achieved by the three bacterial treatments and the TSB treatment (Fig. 3; Fig. S1), there were some differences between these four treatments at some of the five intermediate samplings. For example, 41 days after sowing (T2), lettuce plants inoculated with *P. putida* B21-024 had a higher dry weight and a higher lettuce length than the four other treatments. By contrast, 41 days after sowing (T2), lettuce plants inoculated with the *Pseudomonas* consortium treatment had, on average, fewer leaves than lettuce plants inoculated with the TSB treatment or with the other two bacterial treatments.

At harvest (T6), no significant differences in fresh weight were observed between the five treatments (Fig. 3). Even though the differences were not significant, lettuce plants inoculated with the *Pseudomonas* consortium treatment displayed, on average, an 18% higher fresh weight (1,250 g) than the water-inoculated plants (1,059 g), and 7% higher fresh weight than TSB-inoculated plants (1,164 g). In addition, there were no significant differences between the five treatments regarding lettuce length. Nevertheless, lettuce plants inoculated with *P. protegens* B21-024 or with the *Pseudomonas* consortium displayed a significantly higher dry weight than the water-inoculated lettuce plants (Fig. 3). The *Pseudomonas* consortium treatment led to the highest dry weight increase (+20%). In addition, it was the only treatment significantly different from the TSB treatment for shoot dry weight. Lettuce plants inoculated with one of the three bacterial treatments also had a significantly higher number of leaves than the water-inoculated plants at harvest (Fig. 3). At harvest (T6), the TSB treatment was not significantly different from the water treatment for any of the four plant growth metrics monitored in this study (Fig. 3).

**Fig 4:**
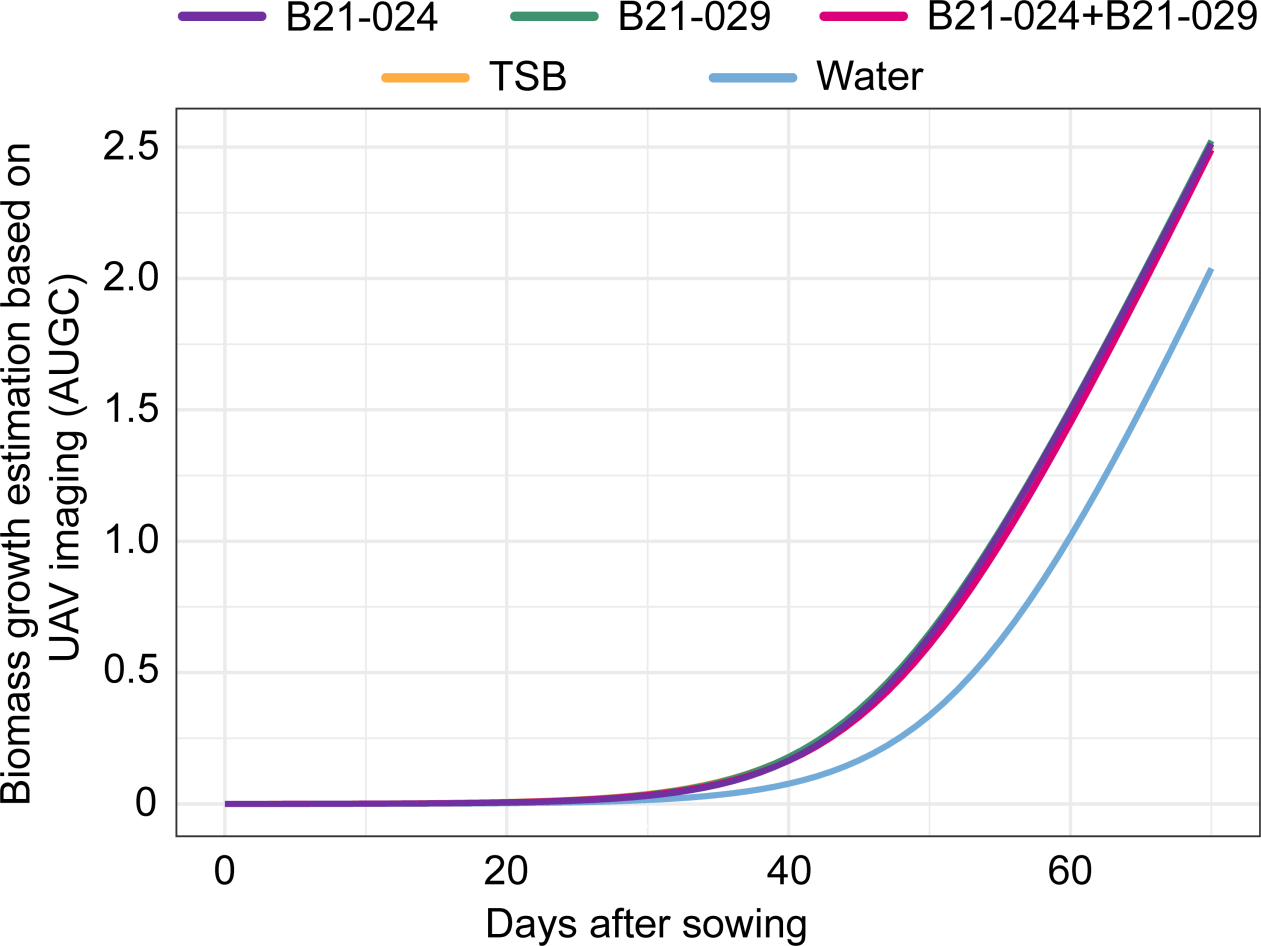
Aboveground biomass estimation based on UAV imaging. The area under the growth curve (AUGC) was obtained by calculating the area under the logistic growth curve modeled from the surface area morphological metric.

In addition to plant growth metrics obtained from weekly manual sampling, biweekly UAV flights mounted with imaging sensors allowed us to closely monitor the growth of each individual lettuce throughout the growing season. UAV imaging is increasingly used to monitor the growth of agricultural crops under field conditions (35) and has been previously used to evaluate the effect of plant growth-promoting rhizobacteria on plant growth (27, 36). Three morphological metrics were calculated for more than 600 lettuce plants per treatment: height, surface area, and volume. The area under the growth curve (AUGC) of the surface area metric was previously chosen as the best morphological metric to monitor biomass accumulation in field-grown lettuce plants (27). The same choice was made in the present study. The AUGC values calculated for each treatment strongly correlated with shoot fresh weight (*r*^2^ = 0.97) and shoot dry weight values (*r*^2^ = 0.95) throughout the growing season (Fig. S2). When comparing AUGC values and shoot fresh/dry weight values for individual lettuce plants at harvest, the correlation was smaller (Fig. S3). The AUGC curves clearly demonstrate the plant growth-promoting effect of the four treatments (B21-024, B21-029, B21-024+B21-029, and TSB) when compared to the water control (Fig. 4). At harvest, plants inoculated with one of the three bacterial treatments or with the TSB treatment had significantly higher AUGC values than water-inoculated plants (*P* value = 0.0017). However, the AUGC values did not allow for discriminating the three bacterial treatments and the TSB treatment from each other.

### Rhizosphere soil population dynamics of *P*. *protegens* B21-024 and *P*. *putida* B21-029

To quantify the abundance of the two *Pseudomonas* strains in rhizosphere soil throughout the growing season (field experiment), two novel strain-specific primer-probe sets were designed. The specificity of the two primer-probe sets was first validated with the DNA obtained from 20 *Pseudomonas* strains belonging to the *P. fluorescens* and *P. putida* groups (Table S1). qPCR amplifications were only observed from the DNA samples corresponding to *P. protegens* B21-024 and *P. putida* B21-29. In addition, no specific amplification was detected in the rhizosphere soil samples collected from lettuce plants inoculated with either TSB or water only. With the specificity of the two primer-probe sets validated, we studied the seasonal dynamics of both strains in rhizosphere soil. In addition, we compared the population dynamics of the *Pseudomonas* strains inoculated alone and in combination.

**Fig 5:**
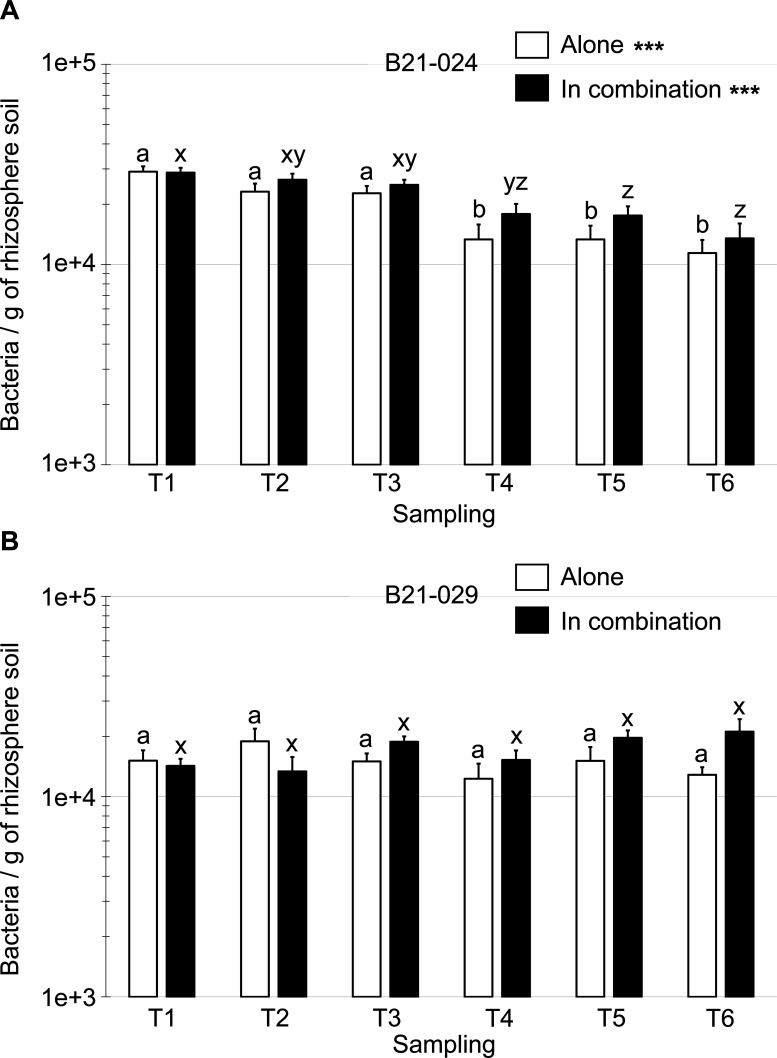
Abundance of *P. protegens* B21-024 (A) and *P. putida* B21-029 (B) in the rhizosphere of lettuce plants throughout the growing season. Each *Pseudomonas* strain was either inoculated alone (white histogram bar) or in combination with the other strain (black histogram bar). Rhizosphere soil samples were collected at six different times during the growing season: 35 (T1), 41 (T2), 49 (T3), 56 (T4), 63 (T5), and 72 (T6) days after sowing. Soil DNA was extracted, and the target sequences were amplified by quantitative PCR using strain-specific primer-probe sets. As each target sequence is present only in one copy in the genome of the bacteria under study, the abundance of the bacteria can be expressed as bacteria per gram of dry rhizosphere soil. Values are presented as means + standard errors. Three asterisks signal *P* values inferior to 0.001 (one-way ANOVA). Treatments with the same letter are not significantly different from each other according to Tukey’s HSD post hoc test (*α* = 0.05).

First, we studied the rhizosphere population dynamics of both strains when inoculated alone (thus excluding for now the rhizosphere soil samples from lettuce plants inoculated with the *Pseudomonas* consortium treatment). *P. protegens* B21-024 was detected in the rhizosphere soil at a population density of 2.9 × 10^4^ bacteria per gram of dry soil at the first sampling time (T1). The population of *P. protegens* B21-024 slightly decreased throughout the growing season, reaching a population of 1.1 × 10^4^ bacteria per gram of dry soil at the end of the growing season (T6). There were significant differences between the sampling times, and the population size of this strain was significantly higher at the first three sampling times. By contrast, the population of *P. putida* B21-029 was extremely stable throughout the growing season, and there were no significant differences between the different sampling times (Fig. 5). The rhizosphere soil population of *P. putida* B21-029 was 1.5 × 10^4^ bacteria per gram of dry soil at the first sampling time (T1). At the end of the growing season (T6), the equivalent of 1.3 × 10^4^ bacteria per gram of dry soil was detected by qPCR.

We then compared the abundance of the two *Pseudomonas* strains in the rhizosphere of lettuce plants when inoculated alone or in combination with the other *Pseudomonas* strain (Fig. 5, Fig. S4). When *P. protegens* B21-024 was inoculated in combination with *P. putida* B21-029, its rhizosphere soil population followed a similar trajectory to when the strain was inoculated alone. Its population size slightly decreased from 2.9 × 10^4^ (T1) to 1.4 × 10^4^ (T6) bacteria per gram of dry soil (Fig. 5). While the dynamic was similar, the population size of *P. protegens* B21-024 when inoculated in combination with *P. putida* B21-029 was significantly higher (*P* value = 0.026) than when this strain was inoculated alone (Fig. S4). When *P. putida* B21-029 was inoculated in combination with *P. protegens* B21-024, its population size was similar to when inoculated alone, with a rhizosphere population size ranging from 1.7 × 10^4^ to 2.1 × 10^4^ bacteria per gram of dry soil. There was also no difference between the population sizes of *P. putida* B21-029, whether the strain was inoculated alone and in combination (Fig. S4).

## DISCUSSION

In this study, we explored the potential of two plant-beneficial *Pseudomonas* strains, *P. protegens* B21-024 and *P. putida* B21-029, to enhance lettuce growth under representative commercial field conditions. The goal was to develop an effective *Pseudomonas* consortium that would efficiently colonize the rhizosphere and synergistically increase lettuce yield.

To favor the establishment of the two *Pseudomonas* strains in the lettuce rhizosphere, fresh TSB medium was added during the preparation of the bacterial inocula. In addition, the bacteria were grown in TSB medium, and while most nutrients were likely used by the bacteria to grow, it is possible that a significant proportion remained. Therefore, a TSB treatment was included in the field experiment to monitor the potential effect of this nitrogen-rich medium on plant growth. It was previously demonstrated that the same TSB treatment promoted the growth of lettuce plants grown under nitrogen-limited conditions but not when using the recommended nitrogen application dose (27). In this study, the TSB treatment promoted the growth of lettuce plants during most of the growing season (T1–T5). Notably, this treatment was significantly different from the water treatment during the first five samplings across all four plant growth metrics (Fig. 3). In addition, biomass estimation based on UAV imaging also identified a plant growth promotion effect associated with this treatment throughout the growing season (Fig. 4).

TSB medium is mainly composed of protein hydrolysates, namely pancreatic digest of casein and papain digest of soybean meal, which are a well-studied class of plant biostimulants (37, 38). Different protein hydrolysates were previously shown to promote the growth (39, 40) and reduce salinity stress in lettuce plants (41). In this study, it is likely that the plant growth promotion effect displayed by TSB originates from its high content in amino acids and peptides, which can be taken up by plant roots as a source of nitrogen (42, 43). Alternatively, the use of TSB might favor the recruitment of indigenous plant-beneficial rhizobacteria, as previously reported in another study (44). Finally, it is also possible that the L-tryptophan amino acids present in casein and soy proteins (45) could be used by indigenous rhizobacteria to produce IAA, as this amino acid is the precursor of IAA in most bacterial biosynthetic pathways (46).

Despite its plant growth-promoting effect, the use of TSB alone could not have accounted for the superior plant growth promotion associated with some bacterial inoculation treatments used in this study. Some bacterial treatments achieved superior plant growth promotion at most of the five intermediate sampling times when compared to the TSB treatment (and the water control) (Fig. 3). Moreover, the TSB treatment was not significantly different from the water control for any of the four plant growth metrics at harvest (T6), while the consortium treatment was significantly superior to the TSB treatment (and the water treatment), and resulted in a significant increase in shoot dry weight (+20%) compared to the water control (Fig. 3). In that regard, the *Pseudomonas* consortium treatment was superior to the *P. protegens* B21-024 inoculation treatment, which was also significantly superior to the water control at harvest (T6) but not significantly different than the TSB treatment. Even though the *Pseudomonas* consortium did not significantly increase lettuce fresh weight at harvest, dry weight is usually considered to be more accurate than fresh weight when evaluating plant growth promotion by plant-beneficial rhizobacteria, mainly because variations in tissue water content between treatments can cause significant data distortion (47).

Single-strain inoculation with *P. protegens* B21-024 previously promoted the growth of lettuce plants under varying nitrogen fertilizer conditions, with dry weight increases ranging from 29.7% to 39.8% depending on the nitrogen fertilization regime (27). In the present study, lettuce plant inoculation with *P. protegens* B21-024 significantly increased shoot dry weight at harvest (T6) by 16% when compared with water-inoculated lettuce plants. However, this treatment was not significantly different from the TSB treatment for shoot dry weight. Several factors could explain this lower performance. First and foremost, the lettuce cultivars used were different, and the one used in the present study “Sun Valley” might be less susceptible to plant growth promotion by rhizobacteria. Several studies have identified a cultivar-specific variation in plant growth promotion by rhizobacteria (48, 49). Environmental conditions between the two growing seasons were also slightly different (the 2024 season was warmer), providing conditions that could potentially be less favorable for the establishment of *Pseudomonas* spp. in 2024. Despite a lower plant growth promotion, this is the second demonstration that inoculation with the DAPG-producing strain *P. protegens* B21-024 significantly increased lettuce shoot dry weight (when compared to the water treatment) under representative commercial field conditions.

Insufficient rhizosphere colonization by plant-beneficial *Pseudomonas* spp. has often been suggested as one of the main reasons why disease suppression and/or plant growth promotion fail under field conditions (8, 50). In this study, the population dynamic of both strains in rhizosphere soil was monitored throughout the growing season to confirm that they were able to maintain moderate-to-high population levels. To this end, strain-specific qPCR primers and probes were successfully developed using a comparative genomic-driven approach. The populations of both *Pseudomonas* strains remained fairly stable throughout the growing season (Fig. 5), with population sizes ranging from 1.1 × 10^4^ to 2.9 × 10^4^ bacteria per gram of dry rhizosphere soil. It is difficult to determine whether such population densities are sufficient to actively promote plant growth under field conditions. By comparison, a population threshold of 10^5^ CFU per gram of fresh root was found to be a prerequisite for the biocontrol of take-all of wheat and Fusarium wilt of radish by plant-beneficial *Pseudomonas* spp. (51, 52). Such population thresholds are often associated with the accumulation of secondary metabolites in the rhizosphere, which directly inhibit pathogen growth (53, 54). To our knowledge, no study has established such population threshold for direct plant growth promotion by *Pseudomonas* spp. In our study, it is highly probable that the populations of both strains were much higher in the peat-based growing medium prior to field transplantation, which enabled a maximum plant growth promotion effect at the beginning of the lettuce growth cycle. In addition, we quantified only the rhizosphere soil populations of *P. protegens* B21-024 and *P. putida* B21-029, and we did not specifically quantify their presence on the root surface (rhizoplane). Therefore, it is likely that the population sizes reported in this study underestimate the total population of both bacteria found in the vicinity of the plant root system. In any case, the population levels attained in this study confirm that both strains successfully colonized the lettuce root system under field conditions over an entire production cycle when inoculated only once before transplantation. Repeated inoculations with *P. protegens* B21-024 and *P. putida* B21-029 should be studied in future experiments to determine if rhizosphere populations may be increased and/or plant-growth promoting effects improved.

One of the most interesting findings of this study was the fact that the rhizosphere population of *P. protegens* B21-024 was significantly higher when co-inoculated with *P. putida* B21-029 than when inoculated alone (Fig. S4). This is surprising since each *Pseudomonas* strain was inoculated at a lower concentration (5 × 10^8^ CFU/mL) in the consortium treatment than when inoculated alone (1 × 10^9^ CFU/mL). Consequently, lower rhizosphere populations were expected. Based on these results, and the absence of inhibition observed between these two strains under *in vitro* conditions (results not shown), it appears that the two *Pseudomonas* strains under study are compatible. Moreover, it is possible that *P. putida* B21-029 could favor the establishment and growth of *P. protegens* B21-024 in the rhizosphere by yet unidentified mechanisms. In previous studies, it was shown that some *Pseudomonas* strains were able to alter the composition of root exudates in favor of microbial growth (55, 56). Analysis of the composition of lettuce root exudates revealed an abundance of amino acids, sugar/sugar alcohols, and organic acids (57). It is possible that *P. putida* B21-029 could degrade some of these compounds into metabolites readily usable by *P. protegens* B21-024. In addition, *P. putida* B21-029 carries a complete type III secretion system (T3SS) cluster in its genome (which is not the case for *P. protegens* B21-024). The role of T3SS in plant-beneficial *Pseudomonas* spp. is not completely understood, but it is hypothesized that this system could help suppress plant immunity and enhance bacterial survival in the soil environment (58, 59). By reducing the plant immune responses, the T3SS of *P. putida* B21-029 could perhaps help *P. protegens* B21-024 to better colonize the lettuce rhizosphere.

In conclusion, we successfully developed a *Pseudomonas* consortium to promote lettuce growth under representative commercial field conditions. Taken together, the results obtained in this study suggest not only that *P. protegens* B21-024 and *P. putida* B21-029 are compatible, but also that they display synergistic activity. In the future, it would be interesting to further study the interaction between both strains to determine whether they directly interact in the rhizosphere, for example, by forming multi-species biofilm, and/or whether they colonize the same niches. This study demonstrates the potential and feasibility of using *Pseudomonas* consortia for leafy greens growth promotion under representative field conditions.

## MATERIALS AND METHODS

### Bacterial strains

Two *Pseudomonas* strains were used in this study, *P. protegens* B21-024 and *P. putida* B21-029. *P. protegens* B21-024 was previously shown to promote the growth of field-grown lettuce plants under varying nitrogen conditions (27). *P. putida* B21-029 promoted the growth of lettuce plants in a preliminary greenhouse experiment (data not shown). Both strains were originally isolated from agricultural soils collected in Southern Quebec (Canada), and their genomes were sequenced in a previous study (60). The strains were routinely grown in TSB medium (BD Difco, Franklin Lakes, NJ) at 25°C for 24 h under agitation (150 rpm).

### Lettuce growth promotion under greenhouse conditions

Romaine lettuce seeds (*Lactuca sativa* L. cv. Sun Valley) were purchased from Norseco (Laval, QC, Canada) and germinated in 72-cell trays filled with a peat-based growing medium (PRO-MIX BX, Premier Tech, Rivière-du-Loup, QC, Canada). Vermiculite was added as a top coating for seed germination. The plants were grown in a climate-controlled greenhouse under a 16-h photoperiod (16 h of light at 21°C followed by 8 h of darkness at 18°C). The seedlings were watered daily and fertilized two times (20 and 24 days after sowing) with 10 mL of a nutrient solution (N100) containing 0.575 g/L of 6-11-31 fertilizer (Master Plant-Prod Inc., Brampton, ON, Canada), 0.425 g/L of 15.5-0-0 fertilizer (Yara International, Oslo, Norway), and 0.13 g/L of Epsom salt (K+S AG, Kassel, Germany). Thirteen days after sowing, each seedling was inoculated by pipetting 10 mL of a bacterial suspension (or water control) at the base of the plant. The following treatments were used: (i) *P. protegens* B21-024 (3 × 10^9^ CFU/mL), (ii) *P. putida* B21-029 (3 × 10^9^ CFU/mL), (iii) combination of B21-024 and B21-029 (5 mL of each bacterial suspension at a concentration of 3 × 10^9^ CFU/mL), and (iv) distilled water (control). The plants were transplanted 27 days after sowing in 2-L plastic pots filled with the same growing medium. The pots were organized in a randomized complete block design with eight replicates per treatment. The plants were fertilized three times (5, 12, and 19 days after transplantation), with 100 mL of a nutrient solution (N200) containing 1.15 g/L of 6-11-31 fertilizer (Master Plant-Prod Inc.), 0.85 g/L of 15.5-0-0 fertilizer (Yara International), and 0.26 g/L of Epsom salt (K+S AG). Fifty-five days after sowing, the plants were harvested, and the aboveground biomass (shoot fresh weight) was measured using a precision scale. The shoots were oven-dried at 65°C for one week (or until completely dried), after which the dry weight was measured.

### Lettuce growth promotion under field conditions

#### Plant material, treatments, and inoculation

Romaine lettuce seeds (*Lactuca sativa* L. cv. Sun Valley) were germinated in 128-cell trays filled with a peat-based growing medium (PRO-MIX BX, Premier Tech). Vermiculite was added as a top coating for seed germination. The seedlings were grown under the same greenhouse conditions as described above. The plants were watered daily and fertilized two times (at 7 and 22 days after sowing), first with the N100 solution and then with the N200 solution. Fifteen days after sowing, the seedling trays were inoculated with one of the five treatments: (i) *P. protegens* B21-024, (ii) *P. putida* B21-029, (iii) combination of B21-024 and B21-029, (iv) fresh TSB medium, and (v) water. The inocula were prepared as previously described (27). Briefly, the bacterial suspensions were first adjusted to an optical density equivalent to 3 × 10^9^ CFU/mL with distilled water. One volume of each bacterial suspension was then mixed with one volume of fresh TSB medium and one volume of distilled water. For the treatment containing the two bacterial strains (B21-024+B21-029), one volume of each bacterial suspension (two volumes in total) was mixed with two volumes of fresh TSB and two volumes of distilled water. The fresh TSB treatment was prepared by mixing two volumes of fresh TSB with one volume of distilled water. The water treatment consisted of distilled water only. The seedling trays were inoculated by soaking them in 5 L of the corresponding inoculum for 5 min. The plants were later transported to the field for transplantation (28 days after sowing).

#### Field site and experimental design

A field experiment was conducted from June 2024 to July 2024 at the Agriculture and Agri-Food Canada experimental farm located in Sainte-Clotilde, QC, Canada (45.163326, −73.673278). Lettuce plantlets were mechanically transplanted in the field 28 days after sowing and harvested 72 days after sowing. During the 44 days the plants were in the field, there was 145 mm of accumulated precipitation, and the average temperature was 20.2°C. The soil was an organic soil representative of nearby commercial fields used for lettuce production. Total soil nitrogen was 25 g/kg. The field was fertilized once the day before transplantation. This was performed according to local fertilization guidelines (61). The entire field received calcium ammonium nitrate 25-0-0 (80 kg N/ha), triple superphosphate 0-46-0 (30 kg P/ha), and potassium chloride 0-0-60 (145 kg K/ha).

A randomized complete block design was used, with eight blocks in total. Each block was subdivided into five plots, and the treatments were randomized between the plots (Fig. 2). Each plot included 80 lettuce plants, planted with a 40 × 30 cm plant spacing. The plants were irrigated only once at planting. Field work included manual weeding and sampling.

#### Field sampling

Lettuce plants were collected six times throughout the growing season, at 35 (T1), 41 (T2), 49 (T3), 56 (T4), 63 (T5), and 72 (T6) days after sowing. One randomly selected lettuce plant per plot was sampled during the five intermediate samplings (T1–T5). Five lettuce plants per plot were harvested at the final sampling (T6). Four growth metrics (shoot fresh weight, lettuce length, and number of leaves) were measured. Lettuce shoots were oven-dried at 65°C for 1 week (or until completely dried), after which the dry weight was measured. All measurements were compiled in a database using the ArcGIS Survey123 (ESRI, Redlands, CA) software installed on a digital tablet (iPad Mini, Apple, Cupertino, CA).

#### Biomass growth estimation using UAV imaging

In addition to manual sampling, UAV imaging was also used to monitor biomass accumulation throughout the growing season. The protocol for crop monitoring leveraged the methodological framework developed by Vigneault et al. (62) for spatiotemporal treatment assessment. Comprehensive aerial surveillance was conducted throughout the complete lettuce development cycle using a DJI Matrice 350 Pro UAV with embedded Zenmuse P1 RGB sensor. Biweekly (twice a week) aerial surveys were performed at precisely calculated altitudes to achieve sub-centimeter spatial resolution—essential for optimal plant segmentation. Flight parameters maintained a minimum 70% image overlap to ensure orthomosaic integrity. Data acquisition accuracy was ensured through a rigorous two-component calibration method: (i) geometric calibration using precisely geolocated ground control points established with RTK GNSS technology (offering millimetric horizontal and centimetric vertical precision); and (ii) photogrammetric processing via Pix4Dmapper software to generate high-precision orthomosaics and digital elevation models at each temporal interval.

For analytical processing, a pre-trained PointRend deep learning architecture was implemented to execute automated segmentation of individual lettuce specimens from the orthomosaic data sets. The extracted morphological parameters (surface area, height, and volumetric measurements) were systematically compiled into a comprehensive database and modeled according to logistic growth functions as per established protocols (62, 63). This approach facilitated quantification of critical growth parameters—initial acceleration, growth rates, deceleration phase, and maximum growth potential—enabling precise assessment of cumulative biomass production and maturity indices across experimental treatments. The AUGC metric, calculated using surface area measurements, was determined to be the optimal parameter for quantifying cumulative treatment effects. This integrated methodology delivered robust, nuanced insights into lettuce growth responses through the synthesis of advanced remote sensing technology and rigorous analytical procedures.

### Detection and quantification of the two *Pseudomonas* strains in rhizosphere soil using qPCR

#### Soil sampling and DNA extraction

At each sampling time, eight rhizosphere soil samples were collected for each treatment (one per block). Rhizosphere soil was manually harvested by collecting 20 mL of soil adhering to the lettuce root system. The samples were kept at −80°C and lyophilized using a FreeZone 6 Liter Console Freeze Dryers (Labconco, Kansas City, MO). DNA extraction was performed using the DNeasy PowerSoil Pro Kit (Qiagen, Hilden, Germany) according to the manufacturer’s instructions. DNA samples were diluted 10-fold with distilled water and kept at −20°C.

#### Primer-probe design

To quantify the abundance of the two bacterial strains in rhizosphere soil throughout the growing season, we designed strain-specific qPCR primers and probes. First, we used a collection of 40 *Pseudomonas* strains to identify, using comparative genomics, potential coding DNA sequence (CDS) targets. The aim was to find CDSs that are only present in one strain and absent from the other 39 strains so that these CDSs might be used as targets for qPCR amplification. Potential CDS targets were identified for *P. protegens* B21-024 and *P. putida* B21-029 using the EDGAR web server (64, 65). The absence of these CDSs in other microorganisms was checked using BLASTn searches against the non-redundant nucleotide (nr/nt) and the whole-genome sequencing (wgs) databases. Two putative CDS targets were identified (one for each strain). Primers and TaqMan minor groove binder (MGB) probes targeting these CDSs were designed with Primer Express 3.0 (Thermo Fisher Scientific, Waltham, MA) using default parameters. The MGB probe was labeled with a 6-FAM reporter dye at the 5′ end, and a nonfluorescent quencher (NFQ) at the 3′ end. The primers and probes used in this study are listed in Table 1. The PCR primers were custom synthesized by Integrated DNA Technologies (Coralville, IA). The TaqMan MGB probes were purchased from Thermo Fisher Scientific.

**TABLE 1:** Primer-probe sets used in this study

Strain	Target gene^*a*^	Primer/probe name	Nucleotide sequence (5′ → 3′)	Amplicon size (bp)	Efficiency (%)
*P*. *protegens* B21-024	LOY29_14745	B21-024_F	GGGTACCTGCGCACTACTTTCT	56	104.5
		B21-024_R	CGCATGCGCGAACAAAC		
		B21-024_P	TTTCGCCGGGAGCG		
*P*. *putida* B21-029	LOY24_13695	B21-029_F	TTTTGAGCATCGACCGAACA	61	103.9
		B21-029_R	CTGAAATAAGCCAGCCGCTATT		
		B21-029_P	CGAGCGAACCTTC		

^*a*^ Both genes are predicted to encode hypothetical proteins.

#### Validation of the primer-probe sets’ specificity

The specificity of the two primer-probe sets was first validated *in silico* with BLASTn against the nr/nt and wgs databases using the amplicon sequences as baits. Primer-BLAST (66) was also used. In addition, the DNA of 20 closely related *Pseudomonas* strains was extracted using the DNeasy UltraClean microbial kit (Qiagen, Toronto, ON, Canada). The strains used are listed in Table S1. The DNA samples were diluted 10-fold and kept at −20°C. These DNA samples were used to validate the specificity of the primer-probe sets, and specific amplification was only detected when using DNA extracted from the two strains under study. The absence of both strains in rhizosphere soil samples from the TSB- and water-inoculated lettuce plants was also validated by qPCR.

#### Standard curves for absolute quantification

For each target CDS, a MiniGene containing the amplicon sequence was purchased from Integrated DNA Technologies. Serial dilutions were performed to generate a standard curve ranging from 10^1^ to 10^8^ amplicon copies per µL. This enabled absolute quantification of the target genes.

#### qPCR

qPCR was performed using an AriaMx real-time PCR system (Agilent, Santa Clara, CA) and the iTaq universal probe supermix kit (Bio-Rad Laboratories, Hercules, CA). Each 10 µL reaction contained 2 µL of template DNA, 5 µL of iTaq universal probe supermix (1×), 0.8 µL of the probe, forward and reverse primers (final concentration of 200 Nmol/L), and 0.6 µL of sterile distilled water. The cycling conditions were 95°C for 2 min, followed by 40 cycles of 95°C for 15 s and 60°C for 1 min. Negative-control reactions were performed during each qPCR run by using sterile distilled water instead of template DNA. Each sample was analyzed in triplicate. Absolute target gene copy numbers were adjusted according to rhizosphere soil dry weight and dilution factor. As each target gene is only present in one copy in the genome of the bacteria under study, the values obtained in gene copy number per gram of dry soil can be converted to bacteria per gram of dry soil.

### Statistical analysis

Statistical analyses were performed in RStudio 2024.09.0 using the package “agricolae” 1.3-7 (Statistical Procedures for Agricultural Research, Felipe de Mendiburu). The non-parametric Friedman test was used to compare treatments in the greenhouse and field experiments. For the field experiment, the five treatments were compared to each other for each sampling time separately. The Fisher’s least significant difference test was used as a post hoc test (*α* = 0.05). To compare the abundance of the two strains in the soil throughout the growing season, one-way analysis of variance (ANOVA) and two-way ANOVA were used. The normality was validated using the Shapiro-Wilk test, and the equality of variance was verified with the Bartlett test (or by plotting residuals). Tukey’s honestly significant difference test was used as a post hoc test (*α* = 0.05).

## SUPPLEMENTAL MATERIAL

Supplemental material spectrum.03509-25-s0001.docx Fig. S1 to S4; Table S1.

ASM does not own the copyrights to Supplemental Material that may be linked to, or accessed through, an article. The authors have granted ASM a non-exclusive, world-wide license to publish the Supplemental Material files. Please contact the corresponding author directly for reuse.

## References

[1] Palleroni NJ. 2005. Genus Pseudomonas, p 323–379. In Brenner D, Krieg NR, Staley JT (ed), Bergey’s manual of systematic bacteriology volume 2: the proteobacteria, part B: the gammaproteobacteria, 2nd ed. Springer, New York.

[2] Girard L, Lood C, Höfte M, Vandamme P, Rokni-Zadeh H, van Noort V, Lavigne R, De Mot R. 2021. The ever-expanding Pseudomonas genus: description of 43 new species and partition of the Pseudomonas putida Group. Microorganisms 9:1766. doi:10.3390/microorganisms9081766 34442845 PMC8401041

[3] Xin X-F, Kvitko B, He SY. 2018. Pseudomonas syringae: what it takes to be a pathogen. Nat Rev Microbiol 16:316–328. doi:10.1038/nrmicro.2018.17 29479077 PMC5972017

[4] Mansfield J, Genin S, Magori S, Citovsky V, Sriariyanum M, Ronald P, Dow M, Verdier V, Beer SV, Machado MA, Toth I, Salmond G, Foster GD. 2012. Top 10 plant pathogenic bacteria in molecular plant pathology. Mol Plant Pathol 13:614–629. doi:10.1111/j.1364-3703.2012.00804.x 22672649 PMC6638704

[5] Haas D, Défago G. 2005. Biological control of soil-borne pathogens by fluorescent Pseudomonads. Nat Rev Microbiol 3:307–319. doi:10.1038/nrmicro1129 15759041

[6] Mercado-Blanco J, Bakker PAHM. 2007. Interactions between plants and beneficial Pseudomonas spp.: exploiting bacterial traits for crop protection. Antonie Van Leeuwenhoek 92:367–389. doi:10.1007/s10482-007-9167-1 17588129

[7] Höfte M. 2021. The use of *Pseudomonas spp*. as bacterial biocontrol agents to control plant disease. In Köhl J, Ravensberg WJ (ed), Microbial bioprotectants for plant disease management https://doi.org/10.19103/AS.2021.0093.11. Burleigh Dodds Science Publishing, Sawston.

[8] Lugtenberg B, Kamilova F. 2009. Plant-growth-promoting rhizobacteria. Annu Rev Microbiol 63:541–556. doi:10.1146/annurev.micro.62.081307.162918 19575558

[9] Zboralski A, Biessy A, Filion M. 2017. Rhizosphere colonization by plant-beneficial *Pseudomonas spp*.: thriving in a heterogeneous and challenging environment, p 197–217. In Singh HB, Sarma BK, Keswani C (ed), Advances in PGPR research. CABI.

[10] Weller DM. 2007. Pseudomonas biocontrol agents of soilborne pathogens: looking back over 30 years. Phytopathology 97:250–256. doi:10.1094/PHYTO-97-2-0250 18944383

[11] Zboralski A, Filion M. 2023. Pseudomonas spp. can help plants face climate change. Front Microbiol 14:1198131. doi:10.3389/fmicb.2023.1198131 37426009 PMC10326438

[12] Loon L. 2007. Plant responses to plant growth-promoting rhizobacteria, p 243–254. In Bakker PAHM, Raaijmakers JM, Bloemberg GV, Höfte M, Lemanceau P, Cooke BM (ed), New perspectives and approaches in plant growth-promoting Rhizobacteria research. Springer, Dordrecht.

[13] Vacheron J, Desbrosses G, Bouffaud M-L, Touraine B, Moënne-Loccoz Y, Muller D, Legendre L, Wisniewski-Dyé F, Prigent-Combaret C. 2013. Plant growth-promoting rhizobacteria and root system functioning. Front Plant Sci 4:356. doi:10.3389/fpls.2013.00356 24062756 PMC3775148

[14] Kang S-M, Radhakrishnan R, Khan AL, Kim M-J, Park J-M, Kim B-R, Shin D-H, Lee I-J. 2014. Gibberellin secreting rhizobacterium, Pseudomonas putida H-2-3 modulates the hormonal and stress physiology of soybean to improve the plant growth under saline and drought conditions. Plant Physiol Biochem 84:115–124. doi:10.1016/j.plaphy.2014.09.001 25270162

[15] Patten CL, Glick BR. 2002. Role of Pseudomonas putida indoleacetic acid in development of the host plant root system. Appl Environ Microbiol 68:3795–3801. doi:10.1128/AEM.68.8.3795-3801.2002 12147474 PMC124051

[16] Oberhansli T, Defago G, Haas D. 1991. Indole-3-acetic acid (IAA) synthesis in the biocontrol strain CHA0 of Pseudomonas fluorescens: role of tryptophan side chain oxidase. J Gen Microbiol 137:2273–2279. doi:10.1099/00221287-137-10-2273 1663150

[17] Glick BR, Nascimento FX. 2021. Pseudomonas 1-Aminocyclopropane-1-carboxylate (ACC) deaminase and its role in beneficial plant-microbe interactions. Microorganisms 9:2467. doi:10.3390/microorganisms9122467 34946069 PMC8707671

[18] Glick BR, Todorovic B, Czarny J, Cheng Z, Duan J, McConkey B. 2007. Promotion of plant growth by bacterial ACC deaminase. CRC Crit Rev Plant Sci 26:227–242. doi:10.1080/07352680701572966

[19] Glick BR. 2014. Bacteria with ACC deaminase can promote plant growth and help to feed the world. Microbiol Res 169:30–39. doi:10.1016/j.micres.2013.09.009 24095256

[20] Miller SH, Browne P, Prigent-Combaret C, Combes-Meynet E, Morrissey JP, O’Gara F. 2010. Biochemical and genomic comparison of inorganic phosphate solubilization in Pseudomonas species. Environ Microbiol Rep 2:403–411. doi:10.1111/j.1758-2229.2009.00105.x 23766113

[21] Rawat P, Das S, Shankhdhar D, Shankhdhar SC. 2021. Phosphate-solubilizing microorganisms: mechanism and their role in phosphate solubilization and uptake. J Soil Sci Plant Nutr 21:49–68. doi:10.1007/s42729-020-00342-7

[22] Park Y-S, Dutta S, Ann M, Raaijmakers JM, Park K. 2015. Promotion of plant growth by Pseudomonas fluorescens strain SS101 via novel volatile organic compounds. Biochem Biophys Res Commun 461:361–365. doi:10.1016/j.bbrc.2015.04.039 25892516

[23] Ryu C-M, Farag MA, Hu C-H, Reddy MS, Wei H-X, Paré PW, Kloepper JW. 2003. Bacterial volatiles promote growth in Arabidopsis. Proc Natl Acad Sci USA 100:4927–4932. doi:10.1073/pnas.0730845100 12684534 PMC153657

[24] Cipriano MAP, Lupatini M, Lopes-Santos L, da Silva MJ, Roesch LFW, Destéfano SAL, Freitas SS, Kuramae EE. 2016. Lettuce and rhizosphere microbiome responses to growth promoting Pseudomonas species under field conditions. FEMS Microbiol Ecol 92:fiw197. doi:10.1093/femsec/fiw197 27660605

[25] Mei C, Zhou D, Chretien RL, Turner A, Hou G, Evans MR, Lowman S. 2023. A potential application of Pseudomonas psychrotolerans IALR632 for lettuce growth promotion in hydroponics. Microorganisms 11:376. doi:10.3390/microorganisms11020376 36838341 PMC9962264

[26] Kangsopa J, Hynes RK, Siri B. 2024. Lettuce seed pelleting with Pseudomonas sp. 31-12: plant growth promotion under laboratory and greenhouse conditions. Can J Microbiol 70:529–537. doi:10.1139/cjm-2024-0071 39116456

[27] Biessy A, Ciotola M, Cadieux M, Deshaies A, Zboralski A, Khun K, Lafond-Lapalme J, Vigneault P, Filion M. 2025. Interplay between nitrogen fertilization and plant growth-promoting rhizobacteria impacts lettuce growth under field conditions. Plant Soil. doi:10.1007/s11104-025-07742-7

[28] Díaz PR, Merlo F, Carrozzi L, Valverde C, Creus CM, Maroniche GA. 2023. Lettuce growth improvement by Azospirillum argentinense and fluorescent Pseudomonas co-inoculation depends on strain compatibility. Agric, Ecosyst Environ, Appl Soil Ecol 189:104969. doi:10.1016/j.apsoil.2023.104969

[29] Liu X, Mei S, Salles JF. 2023. Inoculated microbial consortia perform better than single strains in living soil: a meta-analysis. Agric, Ecosyst Environ, Appl Soil Ecol 190:105011. doi:10.1016/j.apsoil.2023.105011

[30] Kaminsky LM, Trexler RV, Malik RJ, Hockett KL, Bell TH. 2019. The inherent conflicts in developing soil microbial inoculants. Trends Biotechnol 37:140–151. doi:10.1016/j.tibtech.2018.11.011 30587413

[31] Grosskopf T, Soyer OS. 2014. Synthetic microbial communities. Curr Opin Microbiol 18:72–77. doi:10.1016/j.mib.2014.02.002 24632350 PMC4005913

[32] Gross H, Loper JE. 2009. Genomics of secondary metabolite production by Pseudomonas spp. Nat Prod Rep 26:1408–1446. doi:10.1039/b817075b 19844639

[33] Lyng M, Þórisdóttir B, Sveinsdóttir SH, Hansen ML, Jelsbak L, Maróti G, Kovács ÁT. 2024. Taxonomy of Pseudomonas spp. determines interactions with Bacillus subtilis. mSystems 9:e00212–24. doi:10.1128/msystems.00212-24 39254334 PMC11494997

[34] Couillerot O, Combes-Meynet E, Pothier JF, Bellvert F, Challita E, Poirier M-A, Rohr R, Comte G, Moënne-Loccoz Y, Prigent-Combaret C. 2011. The role of the antimicrobial compound 2,4-diacetylphloroglucinol in the impact of biocontrol Pseudomonas fluorescens F113 on Azospirillum brasilense phytostimulators. Microbiology (Reading) 157:1694–1705. doi:10.1099/mic.0.043943-0 21273247

[35] Tsouros DC, Bibi S, Sarigiannidis PG. 2019. A review on UAV-based applications for precision agriculture. Information 10:349. doi:10.3390/info10110349

[36] de Souza AES, Barbosa Júnior MR, de Almeida Moreira BR, da Silva RP, Lemos LB. 2022. UAV multispectral data: a reliable approach for managing phosphate-solubilizing bacteria in common bean. Agronomy 12:2284. doi:10.3390/agronomy12102284

[37] Colla G, Hoagland L, Ruzzi M, Cardarelli M, Bonini P, Canaguier R, Rouphael Y. 2017. Biostimulant action of protein hydrolysates: unraveling their effects on plant physiology and microbiome. Front Plant Sci 8:2202. doi:10.3389/fpls.2017.02202 29312427 PMC5744479

[38] Pasković I, Popović L, Pongrac P, Polić Pasković M, Kos T, Jovanov P, Franić M. 2024. Protein hydrolysates—production, effects on plant metabolism, and use in agriculture. Horticulturae 10:1041. doi:10.3390/horticulturae10101041

[39] Choi S, Colla G, Cardarelli M, Kim H-J. 2022. Effects of plant-derived protein hydrolysates on yield, quality, and nitrogen use efficiency of greenhouse grown lettuce and tomato. Agronomy 12:1018. doi:10.3390/agronomy12051018

[40] Zahra AM, Uthairatanakij A, Laohakunjit N, Jitareerat P, Kaisangsri N, Tira-Umphon A. 2025. Valorization of expired milk into protein hydrolysate as a plant biostimulant: characterization and application on hydroponically grown cos lettuce. Crops 5:56. doi:10.3390/crops5050056

[41] Zuluaga MYA, Monterisi S, Rouphael Y, Colla G, Lucini L, Cesco S, Pii Y. 2023. Different vegetal protein hydrolysates distinctively alleviate salinity stress in vegetable crops: a case study on tomato and lettuce. Front Plant Sci 14:1077140. doi:10.3389/fpls.2023.1077140 36875568 PMC9975731

[42] Näsholm T, Kielland K, Ganeteg U. 2009. Uptake of organic nitrogen by plants. New Phytol 182:31–48. doi:10.1111/j.1469-8137.2008.02751.x 19210725

[43] Farzadfar S, Knight JD, Congreves KA. 2021. Soil organic nitrogen: an overlooked but potentially significant contribution to crop nutrition. Plant Soil 462:7–23. doi:10.1007/s11104-021-04860-w 34720208 PMC8550315

[44] Costa OYA, Chang J, Li J, van Lith W, Kuramae EE. 2024. Unraveling the impact of protein hydrolysates on rhizosphere microbial communities: Source matters. Agric, Ecosyst Environ, Appl Soil Ecol 196:105307. doi:10.1016/j.apsoil.2024.105307

[45] Friedman M. 2018. Analysis, nutrition, and health benefits of tryptophan. Int J Tryptophan Res 11:1178646918802282. doi:10.1177/1178646918802282 30275700 PMC6158605

[46] Tang J, Li Y, Zhang L, Mu J, Jiang Y, Fu H, Zhang Y, Cui H, Yu X, Ye Z. 2023. Biosynthetic pathways and functions of indole-3-acetic acid in microorganisms. Microorganisms 11:2077. doi:10.3390/microorganisms11082077 37630637 PMC10459833

[47] Huang P, de-Bashan L, Crocker T, Kloepper JW, Bashan Y. 2017. Evidence that fresh weight measurement is imprecise for reporting the effect of plant growth-promoting (rhizo)bacteria on growth promotion of crop plants. Biol Fertil Soils 53:199–208. doi:10.1007/s00374-016-1160-2

[48] Chanway C, Nelson L, Holl F. 1988. Cultivar-specific growth promotion of spring wheat (Triticum aestivum L.) by coexistent Bacillus species. Can J Microbiol 34:925–929. doi:10.1139/m88-164

[49] Conn KL, Lazarovits G, Nowak J. 1997. A gnotobiotic bioassay for studying interactions between potatoes and plant growth-promoting rhizobacteria. Can J Microbiol 43:801–808. doi:10.1139/m97-117

[50] Weller DM, Landa BB, Mavrodi OV, Schroeder KL, De La Fuente L, Blouin Bankhead S, Allende Molar R, Bonsall RF, Mavrodi DV, Thomashow LS. 2007. Role of 2,4-diacetylphloroglucinol-producing fluorescent Pseudomonas spp. in the defense of plant roots. Plant Biol (Stuttg) 9:4–20. doi:10.1055/s-2006-924473 17058178

[51] Raaijmakers JM, Weller DM. 1998. Natural plant protection by 2,4-diacetylphloroglucinol-producing Pseudomonas spp. in take-all decline soils. MPMI 11:144–152. doi:10.1094/MPMI.1998.11.2.144

[52] Raaijmakers JM, Leeman M, Oorschot MM, Sluis I, Schippers B, Bakker P. 1996. Dose-response relationships in biological control of fusarium wilt of radish by Pseudomonas spp. Phytopathology 85:1075. doi:10.1094/Phyto-85-1075

[53] Raaijmakers JM, Bonsall RF, Weller DM. 1999. Effect of population density of Pseudomonas fluorescens on production of 2,4-diacetylphloroglucinol in the rhizosphere of wheat. Phytopathology 89:470–475. doi:10.1094/PHYTO.1999.89.6.470 18944718

[54] Mavrodi DV, Mavrodi OV, Parejko JA, Bonsall RF, Kwak YS, Paulitz TC, Thomashow LS, Weller DM. 2012. Accumulation of the antibiotic phenazine-1-Carboxylic acid in the rhizosphere of dryland cereals. Appl Environ Microbiol 78:804–812. doi:10.1128/AEM.06784-11 22138981 PMC3264129

[55] Zhang H, Zheng D, Hu C, Cheng W, Lei P, Xu H, Gao N. 2023. Certain tomato root exudates induced by Pseudomonas stutzeri NRCB010 enhance its rhizosphere colonization capability. Metabolites 13:664. doi:10.3390/metabo13050664 37233705 PMC10220591

[56] Ankati S, Rani TS, Podile AR. 2019. Changes in root exudates and root proteins in groundnut–Pseudomonas sp. interaction contribute to root colonization by bacteria and defense response of the host. J Plant Growth Regul 38:523–538. doi:10.1007/s00344-018-9868-x

[57] Neumann G, Bott S, Ohler MA, Mock H-P, Lippmann R, Grosch R, Smalla K. 2014. Root exudation and root development of lettuce (Lactuca sativa L. cv. Tizian) as affected by different soils. Front Microbiol 5:2. doi:10.3389/fmicb.2014.00002 24478764 PMC3901204

[58] Zboralski A, Biessy A, Filion M. 2022. Bridging the gap: type III secretion systems in plant-beneficial bacteria. Microorganisms 10:187. doi:10.3390/microorganisms10010187 35056636 PMC8780523

[59] Mavrodi DV, Joe A, Mavrodi OV, Hassan KA, Weller DM, Paulsen IT, Loper JE, Alfano JR, Thomashow LS. 2011. Structural and functional analysis of the type III secretion system from Pseudomonas fluorescens Q8r1-96. J Bacteriol 193:177–189. doi:10.1128/JB.00895-10 20971913 PMC3019950

[60] Zboralski A, Biessy A, Ciotola M, Cadieux M, Albert D, Blom J, Filion M. 2022. Harnessing the genomic diversity of Pseudomonas strains against lettuce bacterial pathogens. Front Microbiol 13:1038888. doi:10.3389/fmicb.2022.1038888 36620043 PMC9814014

[61] CRAAQ. 2015. Guide de référence en fertilisation. Québec, Canada Centre de référence en agriculture et agroalimentaire du Québec

[62] Vigneault P, Lafond-Lapalme J, Deshaies A, Khun K, de la Sablonnière S, Filion M, Longchamps L, Mimee B. 2024. An integrated data-driven approach to monitor and estimate plant-scale growth using UAV. ISPRS Open Journal of Photogrammetry and Remote Sensing 11:100052. doi:10.1016/j.ophoto.2023.100052

[63] Go S-H, Lee D-H, Na S-I, Park J-H. 2022. Analysis of growth characteristics of kimchi cabbage using drone-based cabbage surface model image. Agriculture 12:216. doi:10.3390/agriculture12020216

[64] Dieckmann MA, Beyvers S, Nkouamedjo-Fankep RC, Hanel PHG, Jelonek L, Blom J, Goesmann A. 2021. EDGAR3.0: comparative genomics and phylogenomics on a scalable infrastructure. Nucleic Acids Res 49:W185–W192. doi:10.1093/nar/gkab341 33988716 PMC8262741

[65] Blom J, Albaum SP, Doppmeier D, Pühler A, Vorhölter F-J, Zakrzewski M, Goesmann A. 2009. EDGAR: A software framework for the comparative analysis of prokaryotic genomes. BMC Bioinformatics 10:1. doi:10.1186/1471-2105-10-154 19457249 PMC2696450

[66] Ye J, Coulouris G, Zaretskaya I, Cutcutache I, Rozen S, Madden TL. 2012. Primer-BLAST: A tool to design target-specific primers for polymerase chain reaction. BMC Bioinformatics 13:1–11. doi:10.1186/1471-2105-13-134 22708584 PMC3412702

