# Supplementary material for: Single-strain and consortium inoculations with plant-beneficial Pseudomonas spp. promote lettuce growth under field conditions
Source: Microbiol Spectr. 2025 Dec 30;14(2):e03509-25. doi: 10.1128/spectrum.03509-25 (PMC12889054; doi:10.1128/spectrum.03509-25)
Supplement: Supplemental material — Fig. S1 to S4; Table S1. [file spectrum.03509-25-s0001.docx]

# SUPPLEMENTARY MATERIAL


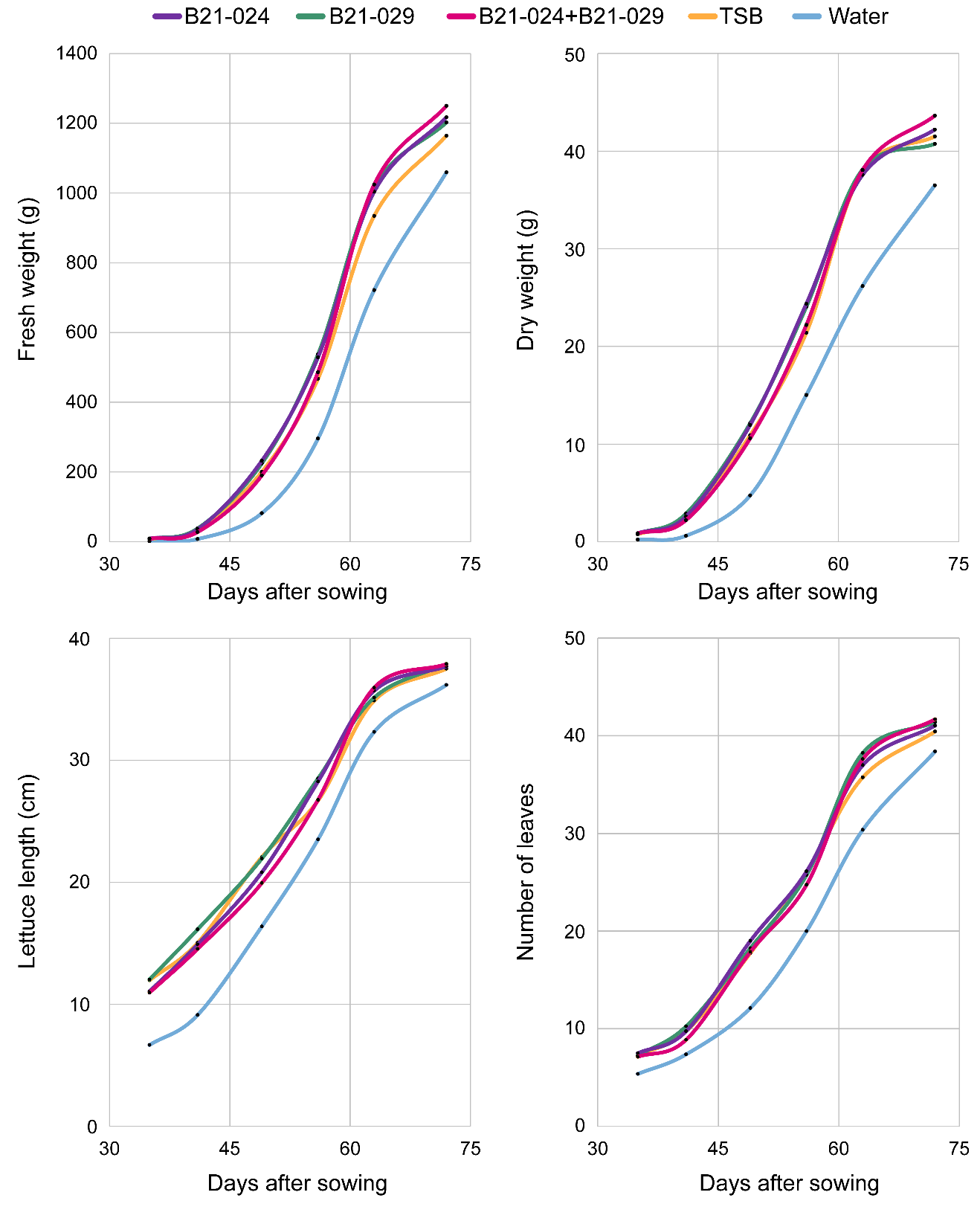


**Figure S1**. Shoot fresh weight, shoot dry weight, lettuce length, and number of leaves throughout the growing season.


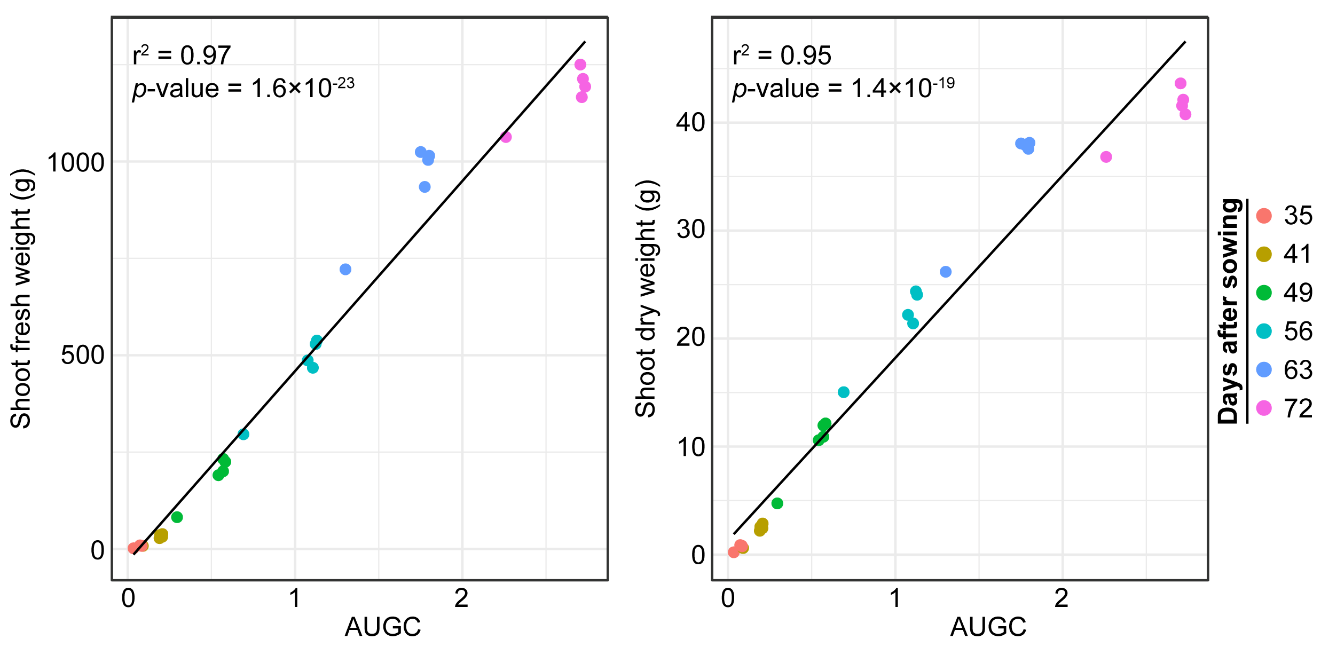


**Figure S2**. Correlation between aboveground biomass (shoot fresh/dry weight) and AUGC values during the growing season.


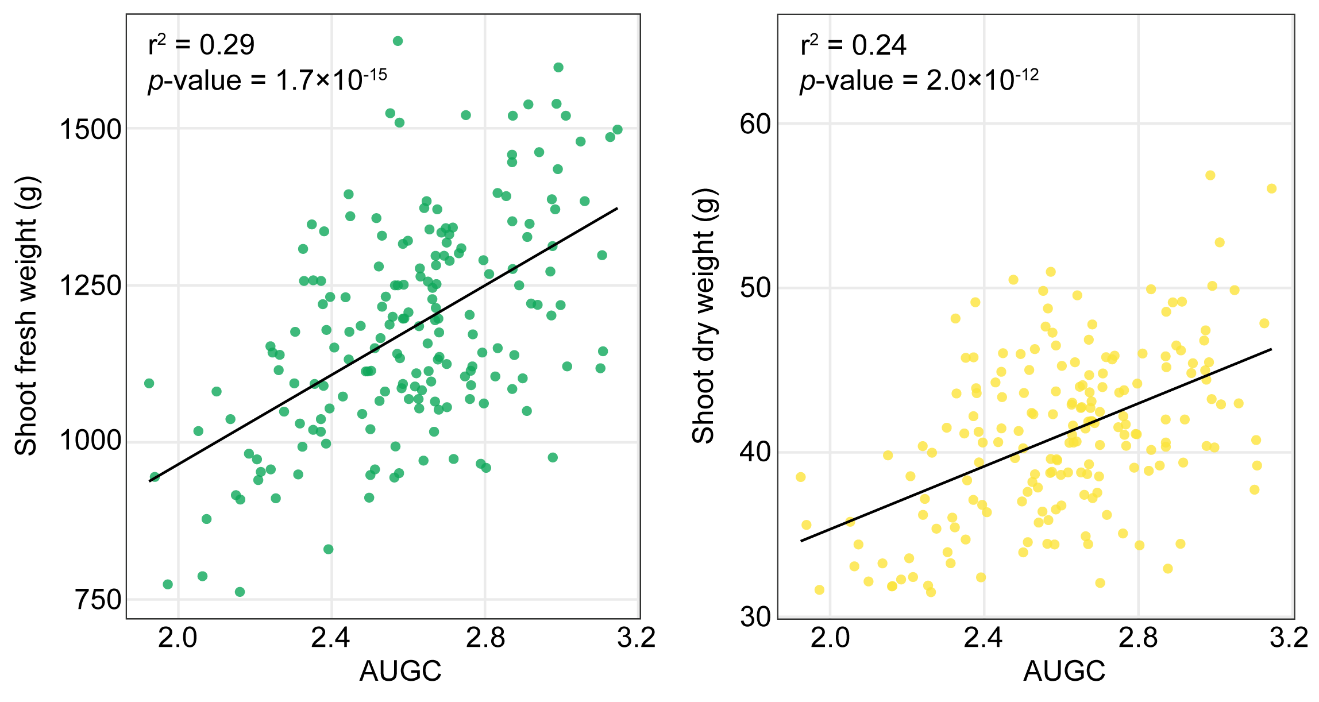


**Figure S3**. Correlation between aboveground biomass (shoot fresh/dry weight) and AUGC values for each individual lettuce at harvest (T6).


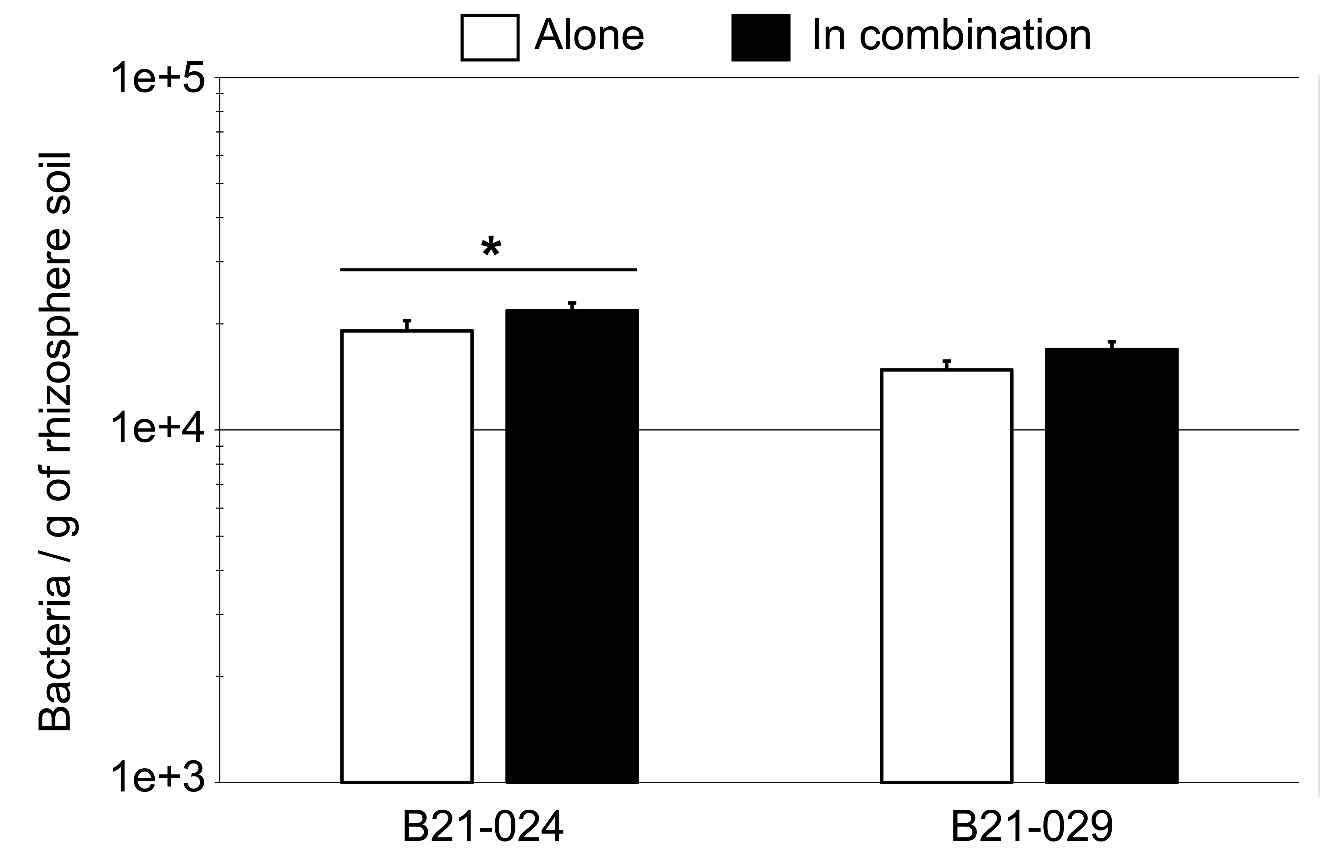


**Figure S4**. Impact of the inoculation approach (alone or in combination) on the abundance of *P*. *protegens* B21-024 and *P*. *putida* B21-029 in rhizosphere soil. The abundance of the two *Pseudomonas* strains was measured with qPCR using strain-specific primer-probe sets. The impact of the inoculation approach on the soil populations was studied using two-way ANOVA. The values presented are the means of the population sizes all time combined (+ standard errors). One asterisk indicates significant difference (*p*-value < 0.05).

**TABLE** **S1**. *Pseudomonas* strains used to validate the specificity of the qPCR primer-probe sets.

| Strain | Group/subgroup | Identification | Genetic relatedness* | Accession number |
| --- | --- | --- | --- | --- |
| **B21-024** | ***P. fluorescens* group/*P. protegens* subgroup** | ***Pseudomonas protegens*** | **100.00** | **CP087189** |
| B21-030 | *P. fluorescens* group/*P. protegens* subgroup | *Pseudomonas protegens* | 98.82 | CP087182 |
| B21-059 | *P. fluorescens* group/*P. protegens* subgroup | *Pseudomonas* sp. | 90.59 | CP087201 |
| CMR5c | *P. fluorescens* group/*P. protegens* subgroup | *Pseudomonas piscis* | 88.09 | CP027705 |
| CMR12a | *P. fluorescens* group/*P. protegens* subgroup | *Pseudomonas sessilinigenes* | 87.83 | CP027706 |
| PA23 | *P. fluorescens* group/*P. chlororaphis* subgroup | *Pseudomonas chl.* subsp*. aureofaciens* | 86.83 | CP008696 |
| PCL1391 | *P. fluorescens* group/*P. chlororaphis* subgroup | *Pseudomonas chl.* subsp*. piscium* | 86.82 | CP027736 |
| M71 | *P. fluorescens* group/*P. chlororaphis* subgroup | *Pseudomonas chl.* subsp*. aurantiaca* | 86.81 | CP027744 |
| 30-84 | *P. fluorescens* group/*P. chlororaphis* subgroup | *Pseudomonas chlororaphis* | 86.80 | CM001559 |
| DSM 50083 | *P. fluorescens* group/*P. chlororaphis* subgroup | *Pseudomonas chl.* subsp*. chlororaphis* | 86.73 | CP027712 |
| B21-058 | *P. fluorescens* group/*P. asplenii* subgroup | *Pseudomonas asplenii* | 83.62 | CP087202 |
| **B21-029** | ***P*. *putida* group** | ***Pseudomonas putida*** | **100.00** | **CP087183** |
| B21-047 | *P*. *putida* group | "*Pseudomonas capeferrum*" | 87.74 | CP087169 |
| B21-031 | *P*. *putida* group | *Pseudomonas kermanshahensis* | 87.62 | CP087181 |
| B21-052 | *P*. *putida* group | *Pseudomonas sichuanensis* | 86.63 | CP087165 |
| B21-027 | *P*. *putida* group | *Pseudomonas sichuanensis* | 86.60 | CP087185 |
| B21-044 | *P*. *putida* group | *Pseudomonas* sp. | 86.25 | CP087172 |
| B21-023 | *P*. *putida* group | *Pseudomonas* sp. | 86.22 | CP087190 |
| B21-036 | *P*. *putida* group | "*Pseudomonas qingdaonensis*" | 84.07 | CP087178 |
| B21-009 | *P*. *putida* group | *Pseudomonas* sp. | 83.99 | CP087199 |
| B21-042 | *P*. *putida* group | *Pseudomonas donghuensis* | 83.95 | CP087174 |
| B21-022 | *P*. *putida* group | *Pseudomonas wadenswilerensis* | 83.94 | CP087191 |
